# Ultrashort pulse laser ablation of dielectrics: Thresholds, mechanisms, role of breakdown

**DOI:** 10.1038/srep39133

**Published:** 2016-12-19

**Authors:** Inam Mirza, Nadezhda M. Bulgakova, Jan Tomáštík, Václav Michálek, Ondřej Haderka, Ladislav Fekete, Tomáš Mocek

**Affiliations:** 1 HiLASE Centre, Institute of Physics ASCR, Za Radnicí 828, 25241 Dolní Břežany, Czech Republic; 2 S.S. Kutateladze Institute of Thermophysics SB RAS, 1 Lavrentyev Ave., 630090 Novosibirsk, Russia; 3 Regional Centre of Advanced Technologies and Materials, Joint Laboratory of Optics of Palacký University and Institute of Physics ASCR, Palacký University, 17. listopadu 12, 771 46 Olomouc, Czech Republic; 4 Joint Laboratory of Optics of Palacký University and Institute of Physics AS CR, 17. listopadu 50a, 772 07 Olomouc, Czech Republic; 5 Institute of Physics ASCR, Na Slovance 1999/2, 18221 Praha, Czech Republic

## Abstract

In this paper, we establish connections between the thresholds and mechanisms of the damage and white-light generation upon femtosecond laser irradiation of wide-bandgap transparent materials. On the example of Corning Willow glass, evolution of ablation craters, their quality, and white-light emission were studied experimentally for 130-fs, 800-nm laser pulses. The experimental results indicate co-existence of several ablation mechanisms which can be separated in time. Suppression of the phase explosion mechanism of ablation was revealed at the middle of the irradiation spots. At high laser fluences, air ionization was found to strongly influence ablation rate and quality and the main mechanisms of the influence are analysed. To gain insight into the processes triggered by laser radiation in glass, numerical simulations have been performed with accounting for the balance of laser energy absorption and its distribution/redistribution in the sample, including bremsstrahlung emission from excited free-electron plasma. The simulations have shown an insignificant role of avalanche ionization at such short durations of laser pulses while pointing to high average energy of electrons up to several dozens of eV. At multi-pulse ablation regimes, improvement of crater quality was found as compared to single/few pulses.

Since the invention of lasers in early 60s, material ablation with various laser sources has been one of the most demanded technologies for drilling micro/nanoholes in various materials, cutting, formation of trenches and other surface and volume relief structures, and material funtionalization^1^,^2^,^3^,^4^,^5^. Although laser processing of materials is much finer as compared to mechanical means, the laser ablation quality is still a matter of research targeted to cleaner crater production with transverse dimensions down to submicrometer size. Among lasers, femtosecond laser sources have been proven to enable production of finest ablation structures due to reduced heat-affected zone^6^,^7^,^8^,^9^. For inorganic dielectrics with low heat conductivity and non-linear light absorption, the fs-laser processing quality can be even higher compared to metals if large temperature and, hence, stress gradients do not lead to crack formation in brittle materials^2^,^3^. Damage can be highly localized when radiation is absorbed by permanent and/or transient photo-induced defect states^10^,^11^. However, due to a complicated interplay between numerous processes initiated by laser action on bandgap materials, the quality of laser processing remains to be a hot topic for research. Laser energy deposition to each material has specific features in terms of damage threshold, efficiency and quality of ablation that is determined by physical material properties.

On the border of 21^st^ century, several historical experiments were performed which gave invaluable insights into the processes upon ultrashort laser ablation of transparent dielectrics and indicated possible routes for ultrafine ablation^12^,^13^,^14^,^15^,^16^,^17^. Those experiments were mostly done for fused silica as an excellent model for in-depth studies due to well-known properties including non-linear behaviour, transient and permanent defects, and wide use in numerous applications^18^. Figure 1 summarises main findings. Stuart *et al*.^15^ identified the damage threshold *F*_th_ for fused silica at 800 nm wavelength by microscopy inspection of the irradiated area. They demonstrated that, at pulse durations *τ*_L_ below ~10 ps, the *F*_th_ value is almost constant with only slight decrease toward shorter pulses down to 100 fs while, with increasing *τ*_L_ above 10 ps, *F*_th_ is increasing as ~*τ*_L_^0.5^ (black dots in Fig. 1). At the same years, several groups made attempts to connect free-electron plasma lightning upon laser excitation with the damage threshold^12^,^13^,^14^. It was shown that appearance of luminous plasma (optical breakdown) could be a good mean to identify *F*_th_ in fused silica at *τ*_L_ ≥ 10 ps. However, at shorter pulses, optical breakdown measurements are contradicting. For femtosecond laser pulses, Du *et al*.^12^,^13^ reported the optical breakdown threshold *F*_OB_ being several times higher than the damage threshold. Varel *et al*.^14^ also found somewhat higher *F*_OB_ compared to the damage threshold at *τ*_L_ in the range of 500 fs–4.5 ps while at shorter pulses they measured the same values for *F*_OB_ and *F*_th_. Note that in ref. 14 the reported *F*_OB_ and *F*_th_ values were a factor of two higher than reported by Stuart *et al*.^15^. At *τ*_L_ ≤ 100 fs, the *F*_OB_ value decreases with shortening pulse duration^13^,^14^ as well as *F*_th_ as shown by Lebugle *et al*.^19^ (empty triangles in Fig. 1). At very short laser pulses, the thresholds for damage^19^ and plasma luminescence^20^ coincide (see Fig. 1).

Lenzner *et al*.^16^,^17^ demonstrated a possibility of production of perfect crater shapes when decreasing the pulse duration to few-cycles (upper panel of Fig. 1). The edges of craters formed by 3-ps and 220-fs pulses are cracked due to high pressure gradients generated by swift heating the irradiated area. Shorter pulses (20 fs, Fig. 1) yield in better crater quality, culminating in the perfect crater produced by 5-fs laser pulse. As for such pulses *F*_OB_ values have not been measured, it can be speculated that the generated free-electron population has no time to become hot enough for transferring much energy to the lattice. As a result, crater formation happens in a “cold ablation” regime without generation of high pressure gradients damaging the crater edges. It should be noted that the “cold ablation” regime is widely accepted to be inherent for IR lasers at *τ*_L_ ≤ 10 ps^21^.

In this paper, we explore the “cold ablation” regime for another glass material, Corning Willow glass (alkali-free borosilicate glass of complex composition)^22^. Compared to fused silica, such glass has lower annealing and melting points and presumably lower band gap. One can expect that the difference in physical properties results in different damage thresholds. However, the same *F*_th_ has been found for our irradiation conditions (Fig. 1). The results of ablation have been studied systematically as a function of laser fluence at 130-fs pules of 800-nm wavelength from the point of view of crater morphology, ablation mechanisms, and free-electron plasma luminosity for single and several laser pulses. To get insight into free-electron plasma formation, modelling has been performed which does not support the concept of “cold ablation”. Although modelling was done for fused silica, the conclusions can be applied to the studied glass in view of similarity in the damage thresholds. Evidences for coexistence of several mechanisms of ablation, including air plasma effects, are discussed.

## Results

Laser ablation of Corning Willow glass^22^ was performed by Ti:Sapphire laser system (Coherent, Legend Elite fs-laser series, 800 nm central wavelength, 130 fs pulse duration, maximum repetition rate of 1 kHz). The main aim was to establish direct links between the three laser-glass interaction aspects, the thresholds for ablation and optical breakdown and ablation quality.

### Single-pulse ablation

**Figure 1:**
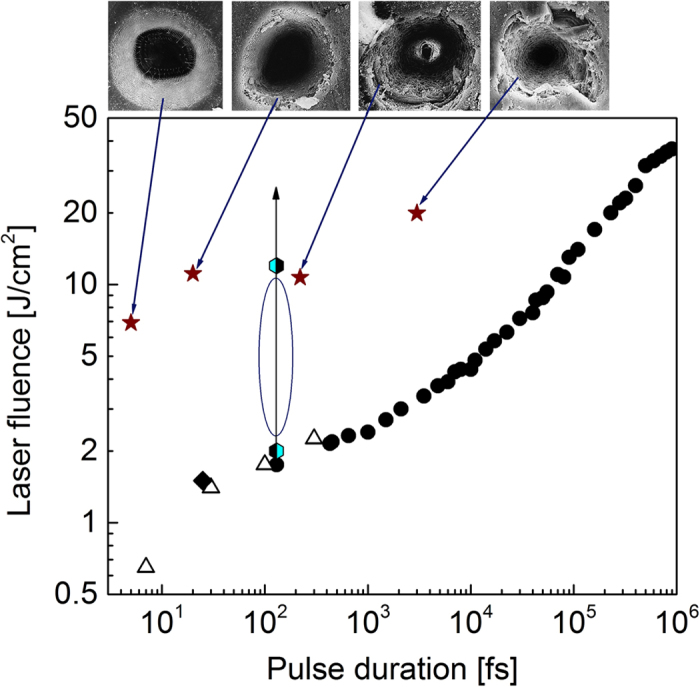
Linking the laser ablation and breakdown thresholds and ablation quality at femto- and picosecond pulse durations for fused silica glass. The damage thresholds are given by black dots (Stuart *et al*.^15^) and open triangles (Lebugle *et al*.^19^). Black rhombic symbol shows the optical breakdown threshold measured at pulse duration of 25 fs by Li *et al*.^20^. At the upper panel, the SEM images of laser-produced craters are introduced from the work by Lenzner *et al*.^17^ with corresponding irradiation regimes shown by stars. Two-colour hexagons indicate the damage (bottom) and breakdown (top) thresholds measured in the present work for Corning’s Willow glass. The regimes studied in this work are shown by vertical arrow pointing up with underlining the conditions of special interest between two thresholds by ellipse.

**Figure 2:**
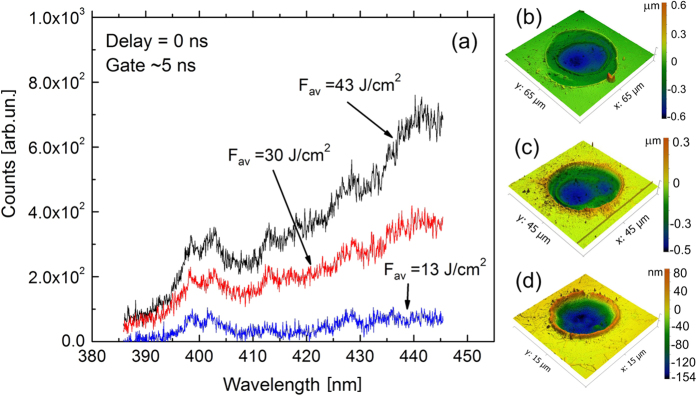
Linking optical emission with crater morphology. (**a**) Early stage optical emission spectra from Willow glass which belongs to a hot electron plasma excited in the sample. Below 13 J/cm^2^ emission is hardly detectable. (**b**–**d**) AFM profiles of the single-shot ablation craters obtained with the average fluences of 59 J/cm^2^ (**b**), 24 J/cm^2^ (**c**), 2.76 J/cm^2^ (**d**).

Figure 2(a) demonstrates the white-light spectra collected from irradiated Willow glass which can be attributed to bremsstrahlung radiation of free electrons generated in the laser-excited sample. The damage threshold was determined at the average fluence of ~2 J/cm^2^ (Fig. 1). At 2.76 J/cm^2^, a well-defined, ~150-nm deep crater with diameter of 10 μm is formed (Fig. 2(d)). The crater is surrounded by a 50-nm high rim whose formation can be attributed to the fact that this material is softer and has a lower melting point compared to fused silica where a rim was not observed^17^.

**Figure 3:**
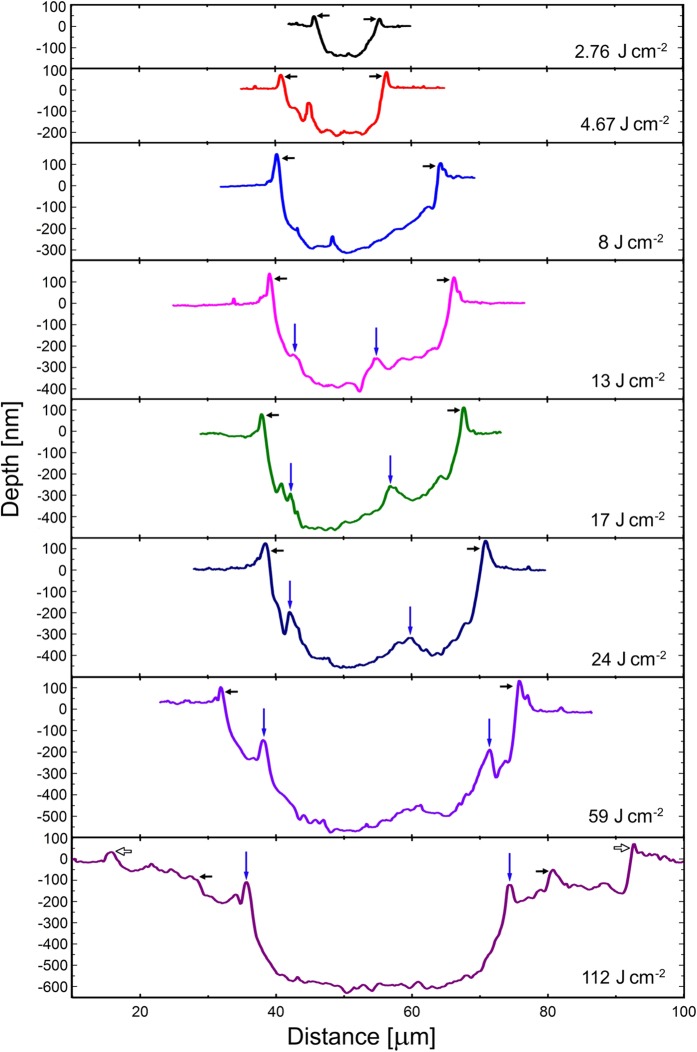
Cross sections of the craters. Primary rims surrounding ablated area are shown by black horizontal arrows. The rims surrounding the secondary craters appearing inside the primary ones are marked by vertical arrows. An outer rim which is observed at energies above 100 J/cm^2^ far beyond the laser irradiated area (marked by empty arrows at the bottom figure) is attributed to the air plasma reemission effect.

Electron plasma luminosity from the sample was detected well above *F*_th_, starting from ~13 J/cm^2^ (*F*_OB_, Fig. 1), that coincides with the data for fused silica obtained in refs 12 and 13. With increasing laser fluence above *F*_OB_, the craters change their forms with clear appearance of a double-rim structure (Fig. 2(b–c)). The sequence of the crater profiles with increasing laser fluence is presented in Fig. 3 (see also Supplementary Fig. S1, in addition to Fig. 2(b–d)).

As expected, the crater diameter and depth are increasing with fluence. At 13 J/cm^2^ the crater is ~25-μm wide and ~400-nm deep. Namely starting from this breakdown threshold, a “secondary crater” appears inside the ablated volume with its “secondary” rim which is well seen at higher fluences (Figs 2(b,c) and 3, and Supplementary Fig. S1). The positions of rims surrounding secondary (internal) craters and the crater bottom geometry indicate that the spatial distribution of laser intensity is asymmetric. It can be due to several reasons such as astigmatism and the output beam divergence. As a result, the M^2^ beam quality factor becomes different for two orthogonal directions across the beam axis with deviating from a diffraction-limited TEM00 laser beam^23^ and yielding a non-circular crater. At high laser fluences, the crater becomes more symmetric (Fig. 3, 112 J/cm^2^). Most plausibly, this can be attributed to air ionization effects^24^,^25^. Upon propagation toward the sample surface, a high-intensity laser beam induces ionization of air molecules that is more efficient in the higher intensity regions of the beam cross section. Before reaching the sample surface, the laser beam can be essentially flattened due to air ionization^24^. Thus, air ionization can lead to beam “self-healing” via smoothing high-intensity imperfectnesses.

Influence of air ionization on crater formation is confirmed by the geometry of craters produced at high laser fluences, of the order of 100 J/cm^2^ and higher (Fig. 3, 112 J/cm^2^). With almost twice increased fluence, the depth of the crater is increasing only slightly (Fig. 3, 59 and 112 J/cm^2^) that can be attributed to enhanced light absorption during beam propagation through air at larger fluences due to multiphoton/tunneling ionization^24^. At fluences ≥100 J/cm^2^ a third, outer rim is formed with the diameter more than 3 times exceeding the beam diameter (1/e^2^). The incident laser energy is too low at the positions between primary and outer rims and, thus, cannot induce material damage. Several mechanisms can contribute to such “matryoshka-like” crater formation with material damage beyond the irradiation spot: (a) air and ablation plasma reemission during recombination of plasmas^24^ (note that air plasma recombines within several nanoseconds after femtosecond laser pulses^26^ while ablation products can produce light emission at a longer timescale upon plume expansion^27^); (b) strong scattering of the tail part of the beam by air plasma produced by the beam front^28^; (c) a strong shock wave generated upon air plasma recombination which travels outward the plasma region and can induce material modification at its contact with the surface^3^,^24^. It should be mentioned that, for our *τ*_L_ at fluences above ~80 J/cm^2^, the beam propagation is affected by the Kerr nonlinearity that provides more effective air ionization before the geometrical focus and, thus, higher scattering of laser light.

**Figure 4:**
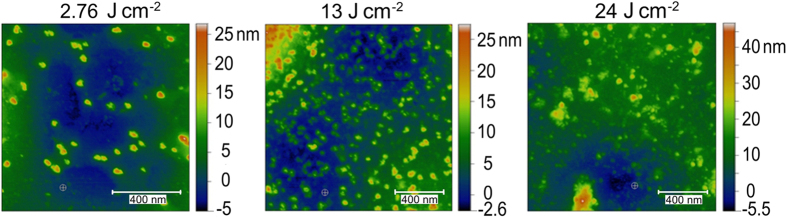
AFM images of the parts of the crater bottoms after single-pulse ablation at different laser fluences. The color scales depict the height of the crater bottom relief with deposited nanoparticles.

Inspection of the craters with AFM (Fig. 4) has shown that the crater bottoms are covered by nanoparticles. Nanoparticles are less abundant at small fluences, being at the same time of larger sizes compared to those at higher fluences. Such morphology of redeposited particles is in line with the phase explosion mechanism of ablation^29^,^30^,^31^,^32^. From the theory of metastable liquid^30^,^32^, it is known that a higher overheating of melt toward the spinodal results in an exponential increase of the rate of vapor nucleation with smaller critical nuclei. This in turn causes disintegration of the overheated melt into vapor and particles of smaller sizes compared to the regimes of lower overheating. As material heating is essentially non-uniform across the irradiation spot, at the spot edges the mechanism of spallation can dominate^29^,^33^, leading to the formation of the rims around the crater assisted by the recoil pressure of the ablation products^34^.

If the origin of the outer rim can be attributed to air ionization effects, appearance of the “internal crater” (or the secondary rim inside the primary crater) is not so clear. One can speculate that this secondary crater formation is conditioned by different mechanisms of ablation dominating at different regions within the laser irradiation spot and developing on different timescales. One of the plausible explanations could be in delayed phase explosion in the middle part of the irradiation spot, due to swift atomization of material in the surface layer of this part (see Fig. 16 in ref. 33). An atomized material layer can temporally suppress the development of explosive vaporization of deeper layers by the recoil force until its expansion and pressure dropping nearby the surface. Coincidence of the secondary rim formation with *F*_OB_ can also indicate the possibility of the Coulomb explosion mechanism of ablation^35^,^36^ from the middle part of the irradiated area where highly-luminous (and hence very hot) electron plasma is created. Photoemission measurements^37^ have revealed superfast electron current from fused silica (electron Coulomb explosion). Such a strong photoemission can also be followed by Coulomb explosion of ions at high velocities, thus assisting in temporal suppression of explosive material emission from deeper layers. Additional discussion of the ablation mechanisms is presented below based on the modelling results.

### Multi-pulse ablation

**Figure 5:**
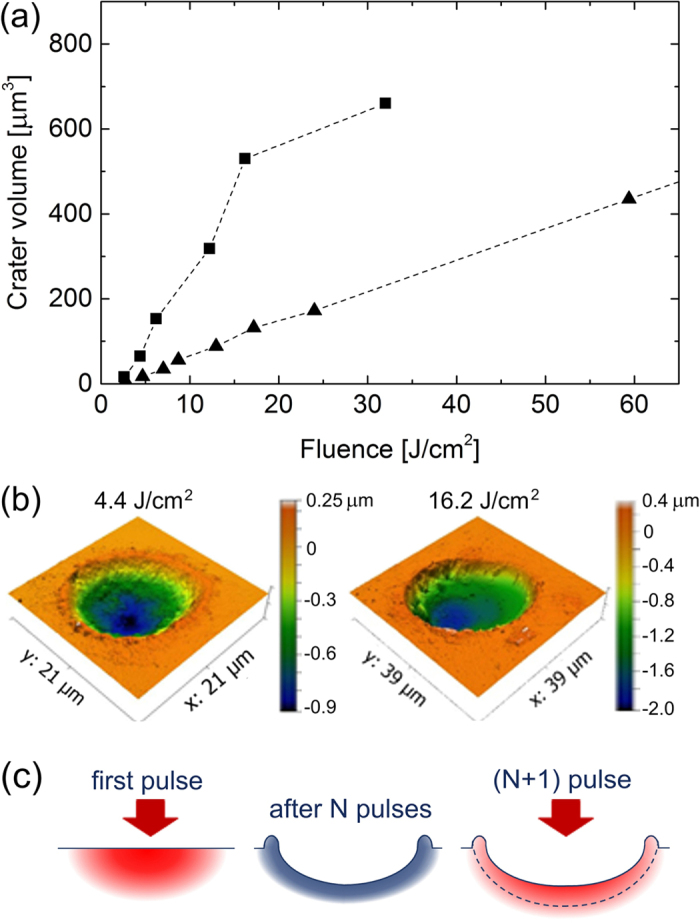
Comparing single- and multi-short laser ablation. (**a**) The crater volume as a function of laser fluence for irradiation with one (triangles) and five (squares) pulses. (**b**) Craters produced by 5 laser pulses at the regimes below (left) and above (right) the breakdown threshold (pulse repetition rate is 10 Hz). (**c**) Schematic illustration of accumulation effects leading to cleaner crater morphology and rim disappearance with number of pulses at high laser fluences (see text).

As “superclean” ablation reported by Lenzner *et al*.^16^,^17^. was achieved in multi-pulse irradiation regime, of high interest is to investigate how the crater quality is changing with applying several pulses. Figure 5 presents the evolution of the ablation volume (a) and crater morphology (b) for the action of 5 successive laser pulses at repetition rate of 10 Hz. At single-pulse irradiation, the crater volume is gradually increasing with laser fluence mainly due to its widening (Figs 2 and 3; see also Supplemental Fig. S1). In the multi-pulse regime, the crater volume increases until the breakdown threshold is reached while at higher fluences some saturation is observed (Fig. 5(a)). Interestingly, the crater deepens but not widens (compare crater diameters for 16.2 J/cm^2^ in Fig. 5 and 17 J/cm^2^ in Fig. 3). Another peculiar feature is that, at multi-pulse irradiation, the crater becomes cleaner with a smaller rim and smoother walls. This feature can be explained by the accumulation effects (defect accumulation; rapid freezing the glass matrix at a high fictive temperature which corresponds to another density and hence to another index of refraction). For the case of volumetric modification, the defect-assisted light absorption can lead to shielding of deeper material layers via creation of a confined heat-affected zone, resulting in decreased laser beam transmission^38^,^39^. A similar effect can be exhibited upon surface ablation as schematically shown in Fig. 5(c). First pulses result in creation of a rim and a strongly modified material layer at the crater bottom and walls (Fig. 5(c), middle). Next pulses are better coupling with the modified material and ablate a thinner layer (depicted by dashed line in Fig. 5(c), right), including the rim consisting from rapidly quenched material melt. The fact that, upon irradiation of fused silica by few-cycle pulses, a well-pronounced rim was observed at single-pulse irradiation^19^ while the rim is not seen at multiple pulse irradiation^16^,^17^, supports the conclusions made above. Note that at low energies, the 5-pulse crater has still rough walls and a well-developed rim (Fig. 5(b), left), suggesting that laser intensity is not enough to produce strong accumulation. This statement is further supported by the evolution of crater depth which grows faster with number of pulses at lower pulse energies (Fig. 5(a)). Also note that crack formation at the crater edges in Willow glass was not found, contrary to fused silica at pulse durations ≥20 fs^17^, that can be attributed to specific material properties such as softening points and ultimate strengths.

### Revisiting the model of surface ablation of transparent dielectrics

Following the laser energy balance^40^, the damage/ablation threshold of bandgap materials at ultrashort laser pulses is determined by levels of the density *n*_*e*_ and the average kinetic energy *E*_*e*_ of free electrons reached upon irradiation. If the *n*_*e*_ value is very high while *E*_*e*_ is well below the material bandgap energy, one may state on “cold ablation” through mainly scissoring the atomic bonds without considerable heating of material matrix. On the contrary, a high average kinetic energy of electrons at relatively low *n*_*e*_ can indicate the regimes of “hot ablation” when the lattice is substantially heated upon electron-lattice thermalization and electron recombination. There are experimental evidences that, in fused silica glass near the damage/ablation threshold at ultrashort laser pulses, *n*_*e*_ ≤ 10^20^ cm^−3^ 41,42,43. On one hand, for very short laser pulses, ~100 fs and shorter, when a noticeable part of the laser energy cannot be transferred to the dielectric matrix during the pulse^44^, such *n*_*e*_ levels suppose very high electron energy to fit the energy balance which would result in glass heating at least above the annealing point^40^. On the other hand, being estimated from pump-probe measurements, the free-electron density represents an averaged value along the laser beam propagation direction^43^ and the local density at the surface layer can be considerably higher. Here we address the questions on the *n*_*e*_ and *E*_*e*_ values via revisiting the model of ultrashort laser excitation of dielectric materials.

The simulations have been performed for fused silica whose physical properties necessary for such modeling are reliable. Willow glass composition is unknown to us, however, it is expected that the glass contains 70–80% SiO_2_ as usual for borosilicate glasses. This plausibly implies formation of the defect states in silicate matrix similar to fused silica. In view of similarity in the damage thresholds of fused silica and Willow glasses (Fig. 1), we consider that the modeling-based conclusions can be applied to the latter material. The details of the model and simulations are presented in Methods and Supplementary S2.

### Simulation results and discussion

**Figure 6:**
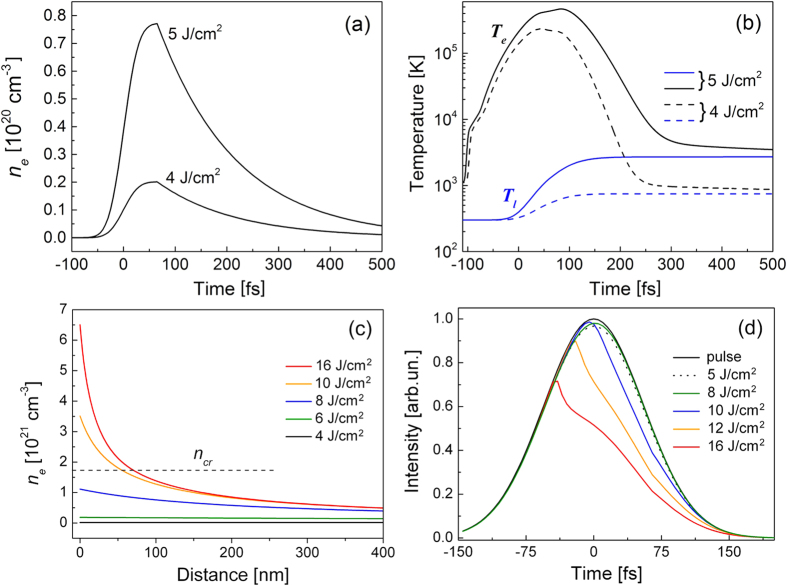
Modeling results on the temporal dynamics of glass excitation and heating. (**a**) Temporal evolution of the electron density on the surface of a fused silica sample for two laser fluences. The simulated fluences of 4 and 5 J/cm^2^ correspond to the average laser fluences, 2 and 2.5 J/cm^2^ respectively. (**b**) The electron and lattice temperature dynamics for the same laser fluences as in (**a**). At 4 J/cm^2^, the lattice remains relatively cold, below the annealing point. At 5 J/cm^2^, the lattice is heated above the melting temperature of 2006 K. (**c**) The spatial profiles of the free-electron density at time moments of 100 fs after the laser pulse maximum. (**d**) Dynamic change in the laser intensity of the beam entering the sample after its partial reflection from the laser-excited surface layer, (1 − *R*(*t*)) × *I*_0_(*t*) where *I*_0_(*t*) and *R*(*t*)) are respectively the incident laser intensity (black solid line) and the time-dependent reflection coeffisient. All data are presented for peak laser fluences.

The results of simulations are presented in Fig. 6. They are obtained for peak laser fluences which are twice larger than the experimentally reported average fluences. Figure 6(a,b) show the temporal evolution of the free-electron density and the electron and lattice temperatures for 4 and 5 J/cm^2^ (average fluences of 2 and 2.5 J/cm^2^). In this range of fluences, the damage threshold is usually identified for fused silica at *τ*_L_ = 130 fs^15^,^17^,^19^,^45^. The final lattice temperature remains below the annealing point at 4 J/cm^2^ while, at 5 J/cm^2^, it exceeds the melting point (Fig. 6(b)). In simulations, *F*_th_ = 2.27 J/cm^2^ is determined at which the annealing point is exceeded and signs of material compaction should be observed upon surface inspection. It is only slightly higher than the measured *F*_th_ (Fig. 1) and, hence, the model reasonably describes the damage threshold. Additionally, the calculated free-electron density fits the measured data for such fluence level^43^ when, according to the present simulations, the free-electron density has still a smooth profile toward the sample depth (Fig. 6(c)). With increasing fluence, strongly inhomogeneous *n*_*e*_ profiles are produced with exceeding the critical plasma density and corresponding formation of a “mirror-like” shielding effect^46^. Dynamics of the laser pulse intensity entering the sample after partial reflection from the excited surface layer is presented in Fig. 6(d), demonstrating that the above-critical free-electron plasma can reduce the effective pulse duration. Note that the instantaneous reflection coefficient was calculated according the multilayer reflectivity model^36^.

While the lattice temperature and the free-electron population look to be reliable, extremely high electron energies (Fig. 6(b)) can raise some doubts. Rather low electron densities generated at fluences near *F*_th_ (Fig. 6(a)) require high electron energies in order the absorbed laser energy could bring material toward the damage. Furthermore, at higher fluences, though the free-electron density increases above 10^20^ cm^−3^, the electron energy also further increases. Thus at 8 J/cm^2^ (average fluence of 4 J/cm^2^), the maximum electron density is already 1.3 × 10^21^ cm^−3^ (Fig. 6(c)) while the electron energy approaches 100 eV that, however, agrees with the energy of photoelectrons observed at similar laser fluences^37^. It should be commented that, in the fluence range applied in simulations (up to 16 J/cm^2^ that corresponds to the average fluence of 8 J/cm^2^), the role of collisional ionization (see Eq. (S5) in Supplementary S2) is rather small. According to modeling, the photoionization process essentially determines electron excitation across the band gap during the laser pulse that is in line with earlier reported results^37^,^47^. However, after the laser pulse, continuing collisional ionization is somewhat suppressing the rate of the free-electron population decay (Fig. 6(a)).

The above-critical “plasma shield” can extend up to 100 nm toward the target (Fig. 6(c)) where the material experiences extremely fast heating toward a hundred thousand Kelvin at 16 J/cm^2^. Such strong heating is sufficient for complete atomization of glass matrix bonds and, additionally, for partial ionization. If to assume that it is feasible, two mechanisms can be responsible for material ablation. As discussed in Section III, in the middle of the irradiation spot where light absorption is largest, Coulomb explosion is highly probable due to energetic electrons escaping from a surface layer and leaving behind a high positive charge^35^,^36^. On the other hand, fast atomization/dissociation of covalently bonded glass matrix has to happen due to rapid lattice heating above the critical thermodynamic temperature^33^. These mechanisms can coexist at high laser intensities though their manifestation can be separated in time with charge-induced emission of ions happening first. As discussed above, swift atomization can be a reason for formation “a crater within the initial crater” via transient suppressing of phase explosion in the hottest middle part of the irradiation spot.

It should be commented that prediction of the ablation depth and crater shape/volume can only be very speculative for the studied conditions. Numerous factors and physical processes must be taken into account: several ablation mechanisms, including phase explosion that implies material ejection in the form of micro/nanoparticles and associated uncertainty in the latent heat of ablation/sublimation of such species; recoil pressure which can either partially suppress or delay the ablation process and, additionally, lead to material relocation within the crater; unknown changes in material properties under heating to extreme thermodynamic conditions, etc. Although modeling results on the spatial profiles of the lattice temperature are in a reasonable agreement with the observed crater depth when accounting for the sublimation energy, we leave this question for further studies.

Extremely high energy of free electrons can result in appearance of luminous plasma lightning that was observed experimentally^12^,^13^,^14^,^16^. Bremsstrahlung radiation can be roughly estimated by a classical expression^24^


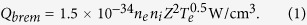


**Figure 7:**
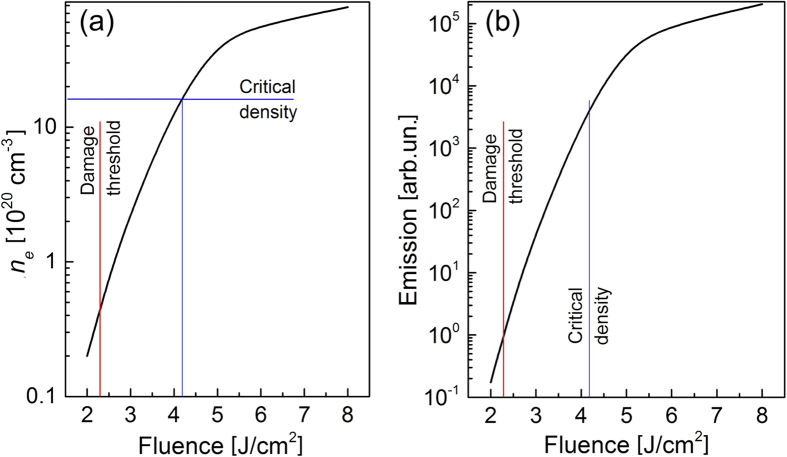
Calculated data on the levels of free-electron density and bremsstrahlung emission. (**a**) The calculated maximum density of free electrons in the center of the irradiation spot on fused silica surface under the action of a 130-fs 800-nm laser pulse as a function of average laser fluence. (**b**) Time-integrated bremsstrahlung emission intensity from the central point of the irradiation spot calculated by Eq. (9) as a function of average laser fluence. The calculated damage threshold and the fluence at which the critical electron density is reached are shown by straight lines. Note that in simulations the fluence was doubled to correspond to the experimental peak fluence. Here the data are presented for average laser fluences.

Here the electron and ion densities (*n*_*i*_ = *n*_*e*_ in the case considered here) are measured in cm^−3^ and the electron temperature is in eV. According to Eq. (1), plasma luminescence is governed mostly by its density and, to a smaller extent, by the electron temperature. Figure 7 presents the evolutions of the maximum free-electron density (a) and the bremsstrahlung signal integrated over time (b). We limited simulations by 16 J/cm^2^ (8 J/cm^2^ of average fluence) for two reasons. First, multiphoton ionization must give way to the tunneling mechanism at such fluences. Second, the role of air ionization already becomes noticeable at such fluences that does not allow to directly compare with the experimental results obtained under the air conditions (the detailed study of the role of air ionization under these particular conditions will be published elsewhere). According to simulations, the critical electron density is achieved in the middle of the irradiation spot at ~4.2 J/cm^2^ (Fig. 7(a)). At such fluence values, the electron population profile is already substantially inhomogeneous (Fig. 6(c)) in the direction to the sample depth and, hence, direct evaluation of the electron density from pump-probe measurements is already problematic^43^.

Due to a swift increase of the free-electron density with fluence, the time-integrated bremsstrahlung emission from the central point of the irradiation spot increases by app. 3.5 orders of magnitude in the fluence range from *F*_th_ to the achieving the critical electron density (Fig. 7(b)). Note that the space integration of bremsstrahlung emission has not been carried out in the present simulations which would further increase the difference in bremsstrahlung signals for low and high fluences. Although some bremsstrahlung emission has to occur as soon as plasma is produced, its detection is determined by the detector sensitivity as noticed by Varel *et al*.^14^. However, we consider that a good concurrence of the thresholds measured in this work with those reported in refs 12, 13, 15 and 19 cannot be occasional, pointing to grasping the physical mechanisms of the studied phenomena.

As a whole, the damage threshold of transparent solids is more deterministic than *F*_OB_ as it is connected with certain values of material physical properties (softening/annealing point, melting point, yield or fracture strength)^48^. If such a value is achieved, the irradiated material changes abruptly its structure/phase and/or massively ablated, e.g. through mechanical fracture. The difference in *F*_th_ for transparent dielectrics at single ultrashort laser pulses of a certain wavelength is mainly conditioned by the difference in the band gap (and, hence, the photo-ionization rate), annealing or melting points, as well as heat capacity. The same damage threshold for fused silica and Willow glass points that the locally absorbed energy in both materials has brought them to the state at least above the annealing point.

Detection of *F*_OB_ is related to bremsstrahlung emission whose intensity is determined by the density and temperature of free-electron “gas”. Hence, some emission can appear as soon as free electrons have been generated. As mentioned above, the breakdown detection is connected with the detection system sensitivity, involving a spectral range of a spectroscopic device or sensitivity of a photodiode. Difference in sensitivity/spectral ranges can be a plausible explanation of the large difference in the measured breakdown thresholds^12^,^13^,^14^.

It should be stressed that the free-electron populations at pico- and femtosecond laser pulses must have different average energies at the same level of laser fluence. This fact inevitably results in a relative shift of the spectral maxima of bremsstrahlung emission that can further affect the accuracy of *F*_OB_ detection. Besides of the spectral shift, a decrease in *F*_OB_ with shortening *τ*_L_ below ~100 fs^13^,^14^ can be attributed to multiphoton intraband transitions (multiphoton absorption of light by free electrons)^49^. The latter process has not been involved in the reported simulations but can become important at increased laser intensities.

At large energies of free electrons reported here, Coulomb explosion looks to be an inevitable mechanism of ablation. However, as soon as a part of energetic free electrons escapes to vacuum, the conduction band of the excited dielectric material cannot be considered anymore as located at near-vacuum level due to the build-up of a high surface potential, preventing further electrons to escape from the surface^50^. Additionally, at high electron densities reached with increasing laser fluence (Fig. 7(a)), the Debye length is extremely small that can suppress electrostatic disintegration of the surface layer.

## Conclusions

In this paper, we have made attempts to establish connections between the thresholds and mechanisms of damage and white-light generation upon femtosecond laser irradiation of wide-bandgap transparent materials. On the example of Corning Willow glass, evolution of ablation craters, their quality, and white-light emission were studied experimentally for 130-fs, 800-nm laser pulses. To gain insight into the processes triggered by laser radiation in glass, numerical simulations have been performed, taking into account bremsstrahlung emission from excited free-electron plasma. The results of this comprehensive study have led to the following conclusions:

- The determined breakdown threshold for Corning Willow glass is considerably higher than the damage threshold in the studied irradiation regimes.
- The evolution of the ablation crater shape and morphology points to co-existence of several ablation mechanisms which can be separated in time. The “crater-inside-crater” shape whose appearance is coincident with *F*_OB_ can be explained by suppression of phase explosion in the middle of the irradiation spot by swiftly atomized external layer of irradiated material.
- At high laser fluences, air ionization strongly influences ablation rate and quality. The main mechanisms of influence can be re-radiation of air plasma upon its recombination, scattering the tail part of the laser beam by air plasma created by the beam front, and shock wave propagating from the region of ionized air, which can etch the sample surface in contact areas.
- Multi-pulse ablation at moderate laser fluences can lead to improvement of crater quality as compared to single pulses.
- Simulations have revealed that the generated free electrons can have very high average energy starting from *F*_th_.
- Simulated temperature dynamics of the lattice indicates that rapid atomization of external target layer can happen at fluences near *F*_OB_ due to swift transfer of energy from the electronic subsystem which is sufficient for scissoring chemical bonds of dielectric matrix.

Obtained results and performed analysis has enabled to clarify a number of effects observed upon glass ablation but also added new questions which call for further studies.

## Methods

### Laser ablation experiments

**Figure 8:**
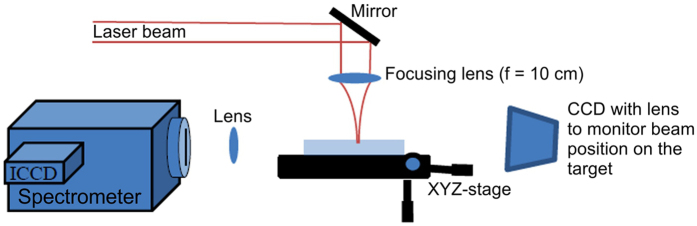
Schematics of the experimental setup.

The scheme of laser ablation experiments is shown in Fig. 8. Laser ablation of glass was performed by Ti: Sapphire fs-laser amplifier (Coherent, Legend Elite fs-laser series, 800 nm) that is capable to produce 4 mJ pulses with pulse duration of ∼130 fs at maximum repetition rate of 1 kHz. The laser energy on the target surface was controlled by a combination of a half-wave plate and a polarizing beam splitter. The Corning Willow glass samples ~100 μm thick were positioned on the XYZ motorized stage. Prior to irradiation, the samples were cleaned in an ultrasonic bath with acetone and isopropanol. The laser beam was focused on the sample surface with a plano-convex lens of 10 cm focal length. The beam waist at focus was measured using a traditional technique^51^ that yielded the beam waist diameter (1/e^2^) at focus of ∼24.5 μm. All experiments have been performed in air under atmospheric conditions.

### Optical emission measurements

The early stage optical emission spectroscopy was performed using a Czerny-Turner spectrometer with a focal length of 163 mm (Andor DH334T-18U-63 ICCD, quantum efficiency of the intensifier in the range of the measured spectra is between 30–47%). The emission was imaged on the spectrometer slit (3 mm × 50 μm) using an achromatic fused silica lens with an overall demagnification of ∼2.5. The emission intensity at the output of spectrometer was recorded as a function of wavelength with the help of a fast ICCD camera (Andor, iStar DH334T ICCD) with a minimum temporal resolution of 3 ns. The ICCD matrix consisted of 1024 × 1024 pixels each with size of 13 × 13 μm^2^. The time delay between the laser pulse and the measurement with the minimum observation gate was adjusted using built-in delay generator of the ICCD. The ICCD was externally triggered by the TTL pulse from the laser system. It was ensured that only emission from free-electron plasma in the sample was recorded, avoiding a contribution from the ablation plasma. The optical emission for a certain laser fluence was recorded for 50 laser shots. The velocity of the translation stage was adjusted to avoid overlapping of individual irradiation spots. The optical breakdown threshold was detected as in the work by Li *et al*.^20^ by change of the slope of the integral emission intensity with increasing laser fluence.

### Analysis of crater morphology

The single-shot ablation craters produced on the target surface at different laser fluences were studied by scanning confocal laser microscope (Olympus LEXT, OLS 3100). The detailed crater morphology was analysed by atomic force microscopy (AFM) operating in tapping mode (Bruker, Dimension Icon ambient AFM in peak force modulation with a tip diameter ≤10 nm). The damage threshold was identified by linear regression of crater volume as a function of fluence from AFM measurements, see Fig. 5. Below this threshold fluence, no visible signs of laser-induced changes were found by microscopy inspection.

### Numerical modeling of laser interaction with fused silica glass

The details of the model equations are given in Supplementary Material (S2). In brief, the one-dimensional model consists of the rate equations describing generation of free electrons and excitons, calculations of the evolving dielectric function of material with swiftly developing electron plasma for determining spatiotemporal dynamics of the coefficients of reflection and absorption, and the heat equations in the form of the two-temperature model (TTM). When considering the TTM, we are aware that, at fs laser pulse timescales, thermal equilibrium within the electron subsystem can occur to be unachievable by the end of the laser pulse^44^. Hence, the free-electron temperature is treated as a measure of the average electron energy. The detailed sets of the model equations can also be found elsewhere^36^,^52^.

For numerical solving the model equations, the finite-difference implicit scheme was used with application of the Thomas algorithm. Numerical grid was regular at the surface layer of 500 nm with the spatial step of 0.5 nm. Deeper to the sample, the spatial step was increased by geometrical progression with the factor of 1.01. The time step of 10^−17^ s secured convergence, accurate solution, and reasonable runtimes.

## Additional Information

**How to cite this article**: Mirza, I. *et al*. Ultrashort pulse laser ablation of dielectrics: Thresholds, mechanisms, role of breakdown. *Sci. Rep.* **6**, 39133; doi: 10.1038/srep39133 (2016).

**Publisher's note:** Springer Nature remains neutral with regard to jurisdictional claims in published maps and institutional affiliations.

## Supplementary Material

Supplementary Information

## References

[b1] Gattass R. R. & Mazur E. Femtosecond laser micromachining in transparent materials. Nat. Photon. 2, 219–225 (2008).

[b2] Bäuerle D. W. Laser Processing and Chemistry. 4th edn, (Springer Berlin Heidelberg, 2011).

[b3] Sugioka K. & Cheng Y. (Ed.) Ultrafast Laser Processing: From Micro- to Nanoscale (Pan Stanford, 2013).

[b4] Thakur P., Tan B. & Venkatakrishnan K. Multi-phase functionalization of titanium for enhanced photon absorption in the vis-NIR region. Sci. Reports 5, 15354 (2015). 10.1038/srep15354 PMC4609984 26477578

[b5] Kerse C. . Ablation-cooled material removal with ultrafast bursts of pulses. Nature, 537, 84–88 (2016). 27409814 10.1038/nature18619

[b6] Chichkov B. N., Momma C., Nolte S., von Alvensleben F. & Tünnermann A. Femtosecond, picosecond and nanosecond laser ablation of solids. Appl. Phys. A 63, 109–115 (1996).

[b7] Sugioka K. & Cheng Y. Ultrafast lasers-reliable tools for advanced materials processing. Light: Sci. Appl. 3, e149 (2014).

[b8] LaHaye N. L., Kurian J., Diwakar P. K., Alff L. & Harilal S. S. Femtosecond laser ablation-based mass spectrometry: An ideal tool for stoichiometric analysis of thin films. Sci. Reports 5, 13121 (2015). 10.1038/srep13121 PMC4541366 26285795

[b9] Malinauskas M. . Ultrafast laser processing of materials: from science to industry, Light: Sci. Appl. 5, e16133, doi: 10.1038/lsa.2016.133 (2016). PMC5987357 30167182

[b10] Barik A. R., Bapna M., Drabold D. A. & Adarsh K. V. Ultrafast light induced unusually broad transient absorption in the sub-bandgap region of GeSe_2_ thin film. Sci. Reports 4, 3686 (2014). 10.1038/srep03686 PMC3890940 24418896

[b11] Batavičiūtė G., Ščiuka M. & Melninkaitis A. Direct comparison of defect ensembles extracted from damage probability and raster scan measurements. J. Appl. Phys. 118, 105306 (2015).

[b12] Du D., Liu X., Korn G., Squier J. & Mourou G. Laser‐induced breakdown by impact ionization in SiO_2_ with pulse widths from 7 ns to 150 fs. Appl. Phys. Lett. 64, 3071–3073 (1994).

[b13] Du D., Liu X. & Mourou G. Reduction of multi-photon ionization in dielectrics due to collisions. Appl. Phys. B 63, 617–621 (1996).

[b14] Varel H. . Laser-induced damage in SiO_2_ and CaF_2_ with picosecond and femtosecond laser pulses. Appl. Phys. A 62, 293–294 (1996).

[b15] Stuart B. C. . Nanosecond-to-femtosecond laser-induced breakdown in dielectrics. Phys. Rev. B 53, 1749–1761 (1996). 10.1103/physrevb.53.1749 9983633

[b16] Lenzner M. . Femtosecond optical breakdown in dielectrics. Phys. Rev. Lett. 80, 4076–4079 (1998).

[b17] Lenzner M., Krausz F., Krüger J. & Kautek W. Photoablation with sub-10 fs laser pulses. Appl. Surf. Sci. 154–155, 11–16 (2000).

[b18] De Jong B. H. W. S., Beerkens R. G. C., van Nijnatten P. A. & Le Bouhis E. Glass, 1. Fundamentals In Ullmann’s Encyclopedia of Industrial Chemistry, 1–54 (Wiley-VCH Verlag GmbH & Co. KGaA), 10.1002/14356007.a12_365.pub3 (2000).

[b19] Lebugle M., Sanner N., Utéza O. & Sentis M. Guidelines for efficient direct ablation of dielectrics with single femtosecond pulses. Appl. Phys. A 114, 129–142 (2014).

[b20] Li M., Menon S., Nibarger J. P. & Gibson G. N. Ultrafast electron dynamics in femtosecond optical breakdown of dielectrics. Phys. Rev. Lett. 82, 2394–2397 (1999).

[b21] Weingarten K. High Energy Picosecond lasers: Ready for prime time. Laser Tech. J. 6, 51–54, doi: 10.1002/latj.200990041 (2009).

[b22] CORNING Willow^®^ Glass. Fact Sheet. http://www.corning.com/media/worldwide/cdt/documents/Willow_2014_fact_sheet.pdf., (Date of access: 15/08/2016) (2016).

[b23] Marshall G. F. & Stutz G. E. (Ed.) Handbook of Optical and Laser Scanning. 2nd edn, 788 (CRC Press, 2011).

[b24] Bulgakova N. M. . Impacts of ambient and ablation plasmas on short- and ultrashort-pulse laser processing of surfaces. Micromachines 5, 1344–1372 (2014).

[b25] Bulgakova N. M., Zhukov V. P., Vorobyev A. Y. & Guo C. Modeling of residual thermal effect in femtosecond laser ablation of metals: role of a gas environment. Appl. Phys. A 92, 883–889 (2008).

[b26] Martirosyan A. E. . Time evolution of plasma afterglow produced by femtosecond laser pulses. J. Appl. Phys. 96, 5450–5455 (2004).

[b27] Bulgakova N. M., Evtushenko A. B., Shukhov Y. G., Kudryashov S. I. & Bulgakov A. V. Role of laser-induced plasma in ultradeep drilling of materials by nanosecond laser pulses. Appl. Surf. Sci. 257, 10876–10882 (2011).

[b28] Klimentov S. M. . Effect of nonlinear light scattering in air on ablation of materials produced by femtosecond laser pulses. Quantum Electron. 32, 433–436 (2002).

[b29] Karim E. T., Wu C. & Zhigilei L. V. Molecular dynamics simulations of laser-materials interactions: General and material-specific mechanisms of material removal and generation of crystal defects In Fundamentals of Laser-Assisted Micro- and Nanotechnologies, Springer Series in Materials Science, Vol. 195 (eds Veiko V. P. & Konov V. I.) 27–49 (Springer International Publishing), doi: 10.1007/978-3-319-05987-7_2 (2014).

[b30] Skripov V. P. Metastable liquids (John Wiley, 1974).

[b31] Miotello A. & Kelly R. Laser-induced phase explosion: new physical problems when a condensed phase approaches the thermodynamic critical temperature. Appl. Phys. A 69, S67–S73 (1999).

[b32] Bulgakova N. M. & Bulgakov A. V. Pulsed laser ablation of solids: transition from normal vaporization to phase explosion. Appl. Phys. A 73, 199–208 (2001).

[b33] Wu C. & Zhigilei L. V. Microscopic mechanisms of laser spallation and ablation of metal targets from large-scale molecular dynamics simulations. Appl. Phys. A 114, 11–32 (2014).

[b34] Körner C., Mayerhofer R., Hartmann M. & Bergmann H. W. Physical and material aspects in using visible laser pulses of nanosecond duration for ablation. Appl. Phys. A 63, 123–131 (1996).

[b35] Stoian R. . Surface charging and impulsive ion ejection during ultrashort pulsed laser ablation. Phys. Rev. Lett. 88, 097603 (2002). 11864053 10.1103/PhysRevLett.88.097603

[b36] Bulgakova N. M., Stoian R., Rosenfeld A., Hertel I. V. & Campbell E. E. B. Electronic transport and consequences for material removal in ultrafast pulsed laser ablation of materials. Phys. Rev. B 69, 054102 (2004).

[b37] Geoffroy G., Duchateau G., Fedorov N., Martin P. & Guizard S. Influence of electron Coulomb explosion on photoelectron spectra of dielectrics irradiated by femtosecond laser pulses. Laser Phys. 24, 086101 (2014).

[b38] Bulgakova N. M. . Pulsed laser modification of transparent dielectrics: what can be foreseen and predicted by numerical simulations? J. Opt. Soc. Am. B 31, C8–C14 (2014).

[b39] Grojo D. . Exciton-seeded multiphoton ionization in bulk SiO_2_. Physical Review B 81, 212301 (2010).

[b40] Bulgakova N. M., Zhukov V. P., Sonina S. V. & Meshcheryakov Y. P. Modification of transparent materials with ultrashort laser pulses: What is energetically and mechanically meaningful? J. Appl. Phys. 118, 233108 (2015).

[b41] Quéré F., Guizard S. & Martin P. Time-resolved study of laser-induced breakdown in dielectrics. EPL 56, 138–144, doi: 10.1209/epl/i2001-00499-9 (2001).

[b42] Mao S. S. . Dynamics of femtosecond laser interactions with dielectrics. Appl. Phys. A 79, 1695–1709 (2004).

[b43] Temnov V. V., Sokolowski-Tinten K., Zhou P., El-Khamhawy A. & von der Linde D. Multiphoton ionization in dielectrics: Comparison of circular and linear polarization. Phys. Rev. Lett. 97, 237403 (2006). 17280244 10.1103/PhysRevLett.97.237403

[b44] Kaiser A., Rethfeld B., Vicanek M. & Simon G. Microscopic processes in dielectrics under irradiation by subpicosecond laser pulses. Phys. Rev. B 61, 11437–11450 (2000).

[b45] Tien A.-C., Backus S., Kapteyn H., Murnane M. & Mourou G. Short-pulse laser damage in transparent materials as a function of pulse duration. Phys. Rev. Lett. 82, 3883–3886 (1999).

[b46] Varkentina N., Sanner N., Lebugle M., Sentis M. & Utéza O. Absorption of a single 500 fs laser pulse at the surface of fused silica: Energy balance and ablation efficiency. J. Appl. Phys. 114, 173105 (2013).

[b47] Wu A. Q., Chowdhury I. H. & Xu X. Femtosecond laser absorption in fused silica: Numerical and experimental investigation. Phys. Rev. B 72, 085128 (2005).

[b48] Chimier B. . Damage and ablation thresholds of fused-silica in femtosecond regime. Phys. Rev. B 84, 094104 (2011).

[b49] Bachau H. . Electron heating through a set of random levels in the conduction band of insulators induced by femtosecond laser pulses. Appl. Phys. A 98, 679–689 (2010).

[b50] Riffe D. M. . Femtosecond thermionic emission from metals in the space-charge-limited regime. J. Opt. Soc. Am. B 10, 1424–1435 (1993).

[b51] Liu J. M. Simple technique for measurements of pulsed Gaussian-beam spot sizes. Opt. Lett. 7, 196–198 (1982). 19710869 10.1364/ol.7.000196

[b52] Bulgakova N. M. . Ultrashort-pulse laser processing of transparent materials: insight from numerical and semi-analytical models. SPIE Proc. 9735, 97350N, 10.1117/12.2217585 (2016).

