# Supplementary material for: Ultrashort pulse laser ablation of dielectrics: Thresholds, mechanisms, role of breakdown
Source: Sci Rep. 2016 Dec 19;6:39133. doi: 10.1038/srep39133 (PMC5171827; doi:10.1038/srep39133)
Supplement: Supplementary Information [file srep39133-s1.pdf]

Supplementary Information

Ultrashort pulse laser ablation of dielectrics: Thresholds, mechanisms, role of breakdown

Inam Mirza[1], Nadezhda M. Bulgakova[1,2*], Jan Tomáštík[4], Václav Michálek [3], Ondřej Haderka[4], Ladislav Fekete[5], Tomáš Mocek[1]

[1]HiLASE Centre, Institute of Physics ASCR, Za Radnicí 828, 25241 Dolní Břežany, Czech Republic. [2]S.S. Kutateladze Institute of Thermophysics SB RAS, 1 Lavrentyev Ave., 630090 Novosibirsk, Russia. [3]Institute of Physics of the Academy of Sciences of the Czech Republic, Joint Laboratory of Optics of Palacký University and Institute of Physics AS CR, 17. listopadu 50a, 772 07 Olomouc, Czech Republic. [4]Regional Centre of Advanced Technologies and Materials, Joint Laboratory of Optics of Palacký University and Institute of Physics ASCR, Palacký University, 17. listopadu 12, 771 46 Olomouc. [5]Institute of Physics ASCR, Na Slovance 1999/2, 18221 Praha, Czech Republic

* e-mail: nadezhda.bulgakova@hilase.cz

Supplementary Information Contents:

S1. Supplementary Figure S1

S2. Supplementary description of the model

S3. Supplementary References

S1. Supplementary Figure S1

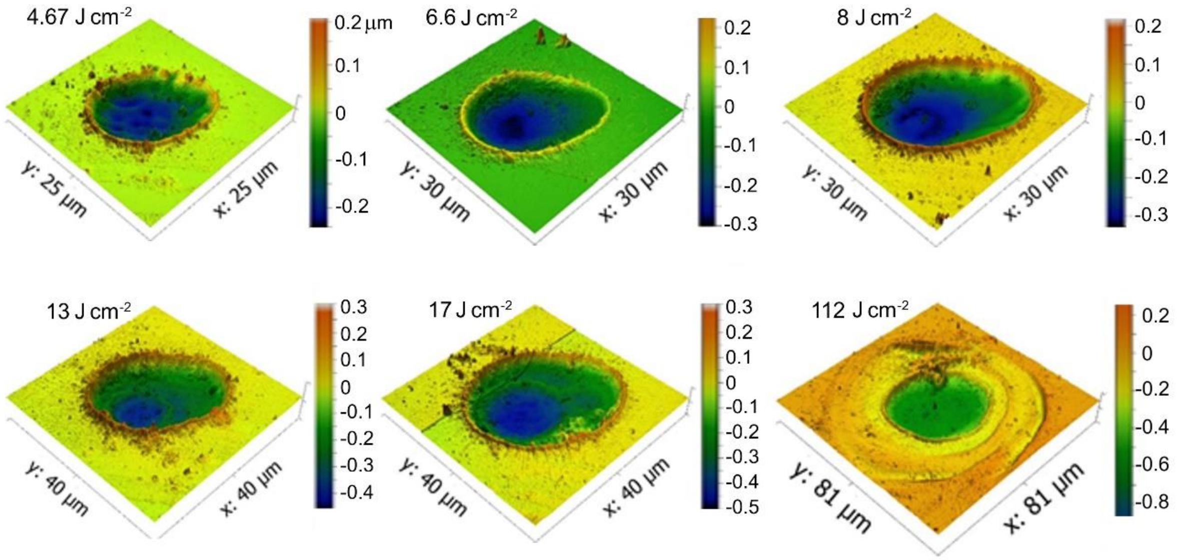

Fig. S1. AFM images of crater profiles obtained by single-shot irradiation showing the evolution of laser ablation features with increasing laser fluence (see also Fig. 2,(b)-(d) in the manuscript). All scales are given in μm.

S2. Supplementary description of the model

The model consists of the rate equations describing generation of free electrons and excitons, calculations of the evolving dielectric function of material with swiftly developing electron plasma for determining spatiotemporal dynamics of the coefficients of reflection and absorption, and the heat equations in the form of the two-temperature model (TTM). When considering the TTM, we are aware that, at fs laser pulse timescales, thermal equilibrium within the electron subsystem can occur to be unachievable by the end of the laser pulse.[1] Hence, the free electron temperature is treated as a measure of the average electron energy. The complete set of equations can be found in [2-4] while here only the main differences and new features introduced into this model are underlined.

The rate equation for the evolution of laser-generated charge carriers was rewritten into two equations describing evolutions of both free electrons and self-trapped excitons (STE) emerging from the free electron trapping process:[5]

$$\frac{\partial n_e}{\partial t} = W_{PI} + W_{PI}^{STE} + W_{av} - \frac{n_e}{\tau_{tr}}, \qquad \text{(S1)}$$

$$\frac{\partial n_{STE}}{\partial t} = -W_{PI}^{STE} + \frac{n_e}{\tau_{tr}} - n_{STE}\frac{1}{\tau_{rec1}}, \qquad \text{(S2)}$$

where n_{STE} is the STE density; τ_{tr} is the characteristic trapping time (taken to be 150 fs).[5] The exciton decay time τ_{rec1} is taken to be 34 ps, disregarding the bi-exponential behavior of exciton decaying where the second decay time is app. 10 times longer ($\tau_{rec2} \approx 338$ ps).[6] Free electron trapping in fused silica occurs into the states located energetically in the bandgap and characterized by the absorption peak at 5.2 eV.[5,7] Hence, four-photon laser excitation of the STEs by the laser beam at 800 nm wavelength can be assumed. At relatively low laser intensities, the photo-ionization rates for valance electrons and the electrons trapped in the STE states were written respectively in the forms[8]

$$W_{PI} = W_{PI0}\left(I / I_*\right)^K (n_{at} - n_e - n_{STE}) / n_{at}, \qquad \text{(S3)}$$

$$W_{PI}^{STE} = W_{PI0}^{STE}\left(I / I_*\right)^{K_{STE}} n_{STE} / n_{at}. \qquad \text{(S4)}$$

Here n_{at} is the atomic density of the unperturbed material; I and I_* are the local laser intensity and the intensity at which the Keldysh parameter $\gamma = 1$ ($I_* = 3.5\times10^{13}$ W/cm^2); $K = 6$ and $K_{STE} = 4$ are the orders of multiphoton ionization of valence and trapped electrons; W_{PI0} and W_{PI0}^{STE} are the multiphoton ionization constants for valence and trapped electrons respectively ($W_{PI0} = 3.7\times10^{34}$ cm$^{-3}\cdot$s^{-1} and $W_{PI0}^{STE} = 2\times10^{37}$ cm$^{-3}\cdot$s^{-1}).[5,9] At fluences $I \geq 0.5\, I_*$, a piecewise linear approximation was built up for W_{PI} which gives the best fit for the photoionization rate reported by Mézel et al.[10]

in different intensity ranges (Perelomov-Popov-Terent'ev model). It is assumed that, during the laser pulse when free electrons are oscillating in the high-intensity wave field, the trapping process is improbable and that the electron trapping can only start at the last quarter of the laser pulse when the beam intensity drops.

The process of collisional multiplication (avalanche) of free electrons under the action of ultrashort laser pulses was intensively discussed in literature.[11-13] One of the most accepted concepts of avalanche considers that the rate of this process can be described as $W_{av} = \alpha_{av} I n_e$ where α_{av} is the avalanche constant.[14,15] This traditional model a priory assumes that kinetic energies of free electrons in the conduction band do not exceed the bandgap energy or, in other words, as soon as a free electron gains kinetic energy which is enough to ionize an atom, the ionization process immediately happens. As a whole, there are opposite viewpoints on the role of avalanche ionization at ultrashort laser pulses. In a number of studies, avalanche is considered as a dominating ionization process even at very short laser pulses[16] while other works report on a negligible role of collisional multiplication of free electron population at similar regimes.[17,18] The photo- and collisional ionization processes together with laser energy absorption by free electrons must fit the energy balance for heating material matrix above at least the annealing point at the damage threshold fluence.[19] This can be possible either (i) at high densities of "cold" free electrons or (ii) with very hot electrons of relatively low density, or (iii) at reasonable values of both n_e and E_e. Recent observation of highly energetic electrons emitted from fused silica subjected to 70-fs laser irradiation[18] suggests rather the second scenario while pointing to a small role of the avalanche process during laser irradiation. It should be admitted that the expression $W_{av} = \alpha_{av} I n_e$ assumes that the rate of collisional multiplication follows the laser beam shape and immediately terminates after the laser pulse termination even if the electrons are still highly energetic. In this

work we have chosen a more physically-grounded avalanche term in the form $W_{av} = \nu_{col} n_e$ where the electron collision frequency ν_{col} is expressed as[20,21]

$$\nu_{col} = \alpha_0 (n_{at} - n_e)\left[(\beta/\pi)^{0.5}(7.5\beta - 1)\exp(-\beta^{-1}) + (3.75\beta^2 - 3\beta + 1)\mathrm{erfc}(\beta^{-0.5})\right], \qquad \text{(S5)}$$

with $\beta = 1.5T_e/E_g$ to be the normalized electron temperature; $\alpha_0 = 1.5$ fs^{-1}. Such a term does not prevent the free electrons to gain any level of kinetic energy and enables to continue collisional ionization after the laser pulse action if the kinetic energy is enough for ionization event.

The attenuation of the laser beam toward material depth is determined by the multiphoton absorption processes and free electron absorption:

$$\frac{\partial}{\partial x} I(x,t) = -6\hbar\omega W_{PI} - 4\hbar\omega W_{PI}^{STE} - a_{\mathrm{ab}}(x,t) I(x,t)\,. \qquad \text{(S6)}$$

The local absorption coefficient α_{ab} as well as the reflection coefficient are calculated from the dielectric function based on the Drude model.[2,3]

The energy balance equations are based on the TTM approach[2-4] where, in the heat flux equation for the electronic subsystem, the energy source term accounts for all the channels of laser energy absorption and redistribution between the electrons, lattice, and the STE states:

$$\frac{\partial E_f}{\partial t} = \left(6\hbar\omega - E_g\right)W_{PI} + \left(4\hbar\omega - E_g^{STE}\right)W_{PI}^{STE} - E_g Q_{\mathrm{av}} + \alpha_{\mathrm{ab}} I\left(x,t\right). \qquad \text{(S7)}$$

An additional energy balance equation is solved, accounting for the energy stored in the STE states:

$$\frac{\partial E_{STE}}{\partial t} = E_g \frac{n_e}{\tau_{tr}} - \left(4\hbar\omega - E_g^{STE}\right)W_{PI}^{STE} - E_g \frac{n_{STE}}{\tau_{rec1}}\,. \qquad \text{(S8)}$$

The first term in the right-hand side of Eq. (S8) assumes that only electrons from the bottom of the conduction band can be trapped and the bandgap energy E_g is stored in both the exciton potential energy E_{PI}^{STE} and the energy of lattice deformation. Upon recombination, the total energy equal to E_g releases to the lattice heating (last term in Eq. (S8) which is also added to the energy equation

for lattice with the positive sign). The energy conservation was satisfied with high accuracy in such forms of the TTM equations.

To account for the electron-lattice coupling, we recall that its characteristic time cannot be longer than the electron trapping time τ_{tr} ~ 150 fs and, hence, lattice heating should occur on extremely short time scale in fused silica glass. The coupling factor was taken similarly to Chimier et al.[22] estimated as $\gamma_{el} = \gamma_0 n_e^{2/3}$ where $\gamma_0 = 0.6\times10^{-3}$ W/(K cm) is a material constant. The system of equations (S1)-(S8) was solved together with the full TTM[2-4,23] and the Drude model for the laser beams of the Gaussian temporal shape of 130-fs duration. Note that, in the present model, the electron energy losses due to bremsstrahlung emission (Eq. (1) of the manuscript) were also added to the electron energy balance term (Eq. (S7)). It has been found that this term does not noticeably contribute to the energy balance.

S3. Supplementary References

1. Kaiser, A., Rethfeld, B., Vicanek, M. & Simon, G. Microscopic processes in dielectrics under irradiation by subpicosecond laser pulses. *Phys. Rev. B* **61**, 11437-11450 (2000).
2. Bulgakova, N. M., Stoian, R., Rosenfeld, A., Hertel, I. V. & Campbell, E. E. B. Electronic transport and consequences for material removal in ultrafast pulsed laser ablation of materials. *Phys. Rev. B* **69**, 054102 (2004).
3. Bulgakova, N. M. *et al.* A general continuum approach to describe fast electronic transport in pulsed laser irradiated materials: The problem of Coulomb explosion. *Appl. Phys. A* **81**, 345-356 (2005).
4. Bulgakova, N. M., Rosenfeld, A., Ehrentraut, L., Stoian, R. & Hertel, I.V. Modeling of electron dynamics in laser-irradiated solids: progress achieved through a continuum approach and future prospects. *SPIE Proc.* **6732**, 673208 (2007).

5. Martin, P. *et al.* Subpicosecond study of carrier trapping dynamics in wide-band-gap crystals. *Phys. Rev. B* **55**, 5799-5810 (1997).

6. Grojo, D. *et al.* Exciton-seeded multiphoton ionization in bulk SiO2. *Phys. Rev. B* **81**, 212301 (2010).

7. Vigouroux, J. P., Duraud, J. P., Le Moel, A., Le Gressus, C. & Griscom, D. L. Electron trapping in amorphous SiO_2 studied by charge buildup under electron bombardment. *J. Appl. Phys.* **57**, 5139-5144 (1985).

8. Bulgakova, N. M. *et al.* Pulsed laser modification of transparent dielectrics: what can be foreseen and predicted by numerical simulations? *J. Opt. Soc. Am. B* **31**, C8-C14 (2014).

9. Couairon, A., Sudrie, L., Franco, M., Prade, B. & Mysyrowicz, A. Filamentation and damage in fused silica induced by tightly focused femtosecond laser pulses. *Phys. Rev. B* **71**, 125435 (2005).

10. Mézel, C., Bourgeade, A. & Hallo, L. Surface structuring by ultrashort laser pulses: A review of photoionization models. *Phys. Plasmas* **17**, 113504 (2010).

11. Schaffer, C. B., Brodeur, A. & Mazur, E. Laser-induced breakdown and damage in bulk transparent materials induced by tightly focused femtosecond laser pulses. *Meas. Sci. Technol.* **12**, 1784–1794 (2001).

12. Gattass, R. R. & Mazur, E. Femtosecond laser micromachining in transparent materials. *Nat. Photon.* **2**, 219–225 (2008).

13. Tien, A.-C., Backus, S., Kapteyn, H., Murnane, M., & Mourou, G. Short-pulse laser damage in transparent materials as a function of pulse duration. *Phys. Rev. Lett.* **82**, 3883-3886 (1999).

14. Epifanov, A. S. Avalanche ionization in solid transparent dielectrics induced by strong laser radiation pulses. *Sov. Phys. JETP* **35**, 897-903 (1975).

15. Stuart, B. C., Feit, M. D., Rubenchik, A. M., Shore, B. W. & Perry, M. D. Laser-induced damage in dielectrics with nanosecond to subpicosecond pulses. *Phys. Rev. Lett.* **74**, 2248-2251 (1995).

16. Du, D., Liu, X., & Mourou, G. Reduction of multi-photon ionization in dielectrics due to collisions. *Appl. Phys. B* **63**, 617-621 (1996).

17. Wu, A. Q., Chowdhury, I. H. & Xu, X. Femtosecond laser absorption in fused silica: Numerical and experimental investigation. *Phys. Rev. B* **72**, 085128 (2005).

18. Geoffroy, G., Duchateau, G., Fedorov, N., Martin, P. & Guizard, S. Influence of electron Coulomb explosion on photoelectron spectra of dielectrics irradiated by femtosecond laser pulses. *Laser Phys.* **24**, 086101 (2014).

19. Bulgakova, N. M., Zhukov, V. P., Sonina, S.V. & Meshcheryakov, Y. P. Modification of transparent materials with ultrashort laser pulses: what is energetically and mechanically meaningful?. *J. Appl. Phys.* **118**, 233108 (2015).

20. Peñano, J. R., Sprangle, P., Hafizi, B., Manheimer, W. & Zigler, A. Transmission of intense femtosecond laser pulses into dielectrics. *Phys. Rev. E* **72**, 036412 (2005).

21. Mézel, C. *et al.* Formation of nanocavities in dielectrics: A self-consistent modeling. *Phys. Plasmas* **15**, 093504 (2008).

22. Chimier, B. *et al.* Damage and ablation thresholds of fused-silica in femtosecond regime. *Phys. Rev. B* **84**, 094104 (2011).

23. Bulgakova, N. M. *et al.* Ultrashort-pulse laser processing of transparent materials: insight from numerical and semi-analytical models. *Proc. SPIE* **9735**, 97350N (2016).
